# Expired Dialysate, Unexpected Pathogen: First Reported Case of *Streptomyces Olivaceus* Peritonitis in a Peritoneal Dialysis Patient

**DOI:** 10.34067/KID.0000001221

**Published:** 2026-04-09

**Authors:** Talerngsak Kanjanabuch, Tanittha Chatsuwan, Piyaporn Towannang, Dhammika Leshan Wannigama, Pongpratch Puapatanakul, Chusana Suankratay

**Affiliations:** 1 Division of Nephrology, Department of Medicine, Faculty of Medicine, Chulalongkorn University, Bangkok, Thailand; 2 Center of Excellence in Kidney Metabolic Disorders, Faculty of Medicine, Chulalongkorn University, Bangkok, Thailand; 3 Peritoneal Dialysis Excellent Center, King Chulalongkorn Memorial Hospital, Bangkok, Thailand; 4 Trinity Center-ISN Interventional Nephrology Training Center, Bangkok, Thailand; 5 Department of Microbiology, Faculty of Medicine, Chulalongkorn University, King Chulalongkorn Memorial Hospital, Thai Red Cross Society, Bangkok, Thailand; 6 Center of Excellence in Antimicrobial Resistance and Stewardship, Faculty of Medicine, Chulalongkorn University, Bangkok, Thailand; 7 Nursing Department, King Chulalongkorn Memorial Hospital, Thai Red Cross Society, Bangkok, Thailand; 8 Department of Infectious Diseases and Infection Control, Yamagata Prefectural Central Hospital, Yamagata, Japan; 9 Faculty of Medicine, Center of Excellence in Antimicrobial Resistance and Stewardship Research, Chulalongkorn University, Bangkok, Thailand; 10 School of Medicine, Faculty of Health and Medical Sciences, The University of Western Australia, Nedlands, Western Australia, Australia; 11 Biofilms and Antimicrobial Resistance Consortium of ODA Receiving Countries, The University of Sheffield, Sheffield, United Kingdom; 12 Pathogen Hunter's Research Team, Department of Infectious Diseases and Infection Control, Yamagata Prefectural Central Hospital, Yamagata, Japan; 13 Department of Infectious Diseases, Faculty of Medicine, Yamagata University and Yamagata University Hospital, Yamagata, Japan; 14 Division of Infectious Diseases, Department of Medicine, Faculty of Medicine, Chulalongkorn University, Bangkok, Thailand

**Keywords:** peritoneal dialysis

## Abstract

Peritoneal dialysis (PD)–associated peritonitis is most commonly caused by skin or enteric organisms, while environmental actinomycetes are rarely implicated. *Streptomyces* species are ubiquitous soil bacteria but are exceedingly uncommon human pathogens, with no prior reports of PD-associated peritonitis confirmed by molecular methods. We report a case of *Streptomyces olivaceus* peritonitis in a 43-year-old man receiving automated PD for 7 months, who presented with abdominal pain and cloudy effluent following the use of an expired dialysate bag. Initial Gram stain revealed filamentous Gram-positive bacilli, but routine cultures were negative, and matrix-assisted laser desorption/ionization-time of flight mass spectrometry misidentified the organism. Because of persistent symptoms, the PD catheter was removed, and subsequent culture demonstrated dry, wrinkled, chalky colonies. Definitive identification was achieved by 16S ribosomal RNA gene sequencing, showing 100% identity with *S*. *olivaceus*. The patient was treated with prolonged antimicrobial therapy and achieved complete clinical recovery. *Post hoc* inspection of the expired dialysate revealed visible defects and polymicrobial contamination, supporting a plausible environmental source. This case highlights key diagnostic challenges in atypical peritonitis, including limitations of conventional microbiologic methods and the critical role of molecular diagnostics. It also underscores the potential risk associated with expired PD supplies, particularly in decentralized or resource-limited settings, and supports the need for enhanced patient-level inspection and supply chain safeguards to prevent similar events.

## Introduction

Peritoneal dialysis (PD)–associated peritonitis remains a clinical challenge in patients with kidney failure, typically caused by skin or enteric organisms.^1^ Environmental actinomycetes, such as *Nocardia*, are less frequently implicated.^2^ *Streptomyces*, a filamentous Gram-positive soil bacterium known for antibiotic biosynthesis, is an exceptionally rare human pathogen, with only two prior cases of peritonitis—neither involving PD nor confirmed by molecular methods.^3–5^

No previous reports have identified a microbiologically confirmed case of PD peritonitis associated with the use of expired dialysate. Expired solutions may compromise sterility through microleaks or packaging degradation and undergo chemical instability, leading to endotoxin accumulation or glucose degradation products, which have been associated with both infectious and sterile peritonitis.^6–9^

We report the first case of *Streptomyces olivaceus* PD-associated peritonitis, temporally associated with the use of an expired dialysate bag, confirmed by 16S ribosomal RNA (rRNA) gene sequencing following misidentification by matrix-assisted laser desorption/ionization-time of flight (MALDI-TOF) mass spectrometry. This case highlights diagnostic challenges in culture-negative peritonitis and the potential risks associated with compromised dialysate integrity.

## Case Description

A 43-year-old Buddhist monk with kidney failure of unknown etiology, receiving automated PD (2L×4 nightly exchanges) for 7 months, presented on Day 0 (July 24, 2025) with diffuse abdominal pain and cloudy PD effluent (PDE). He had no history of peritonitis. His medical history included treated pulmonary tuberculosis, chronic hepatitis C, hypertension, and paroxysmal atrial tachycardia. He had previously undergone arteriovenous graft removal for recurrent infections caused by methicillin-resistant *Staphylococcus aureus*, followed by a 1-month course of intravenous vancomycin (500 mg every 3 days) and linezolid (600 mg every 12 hours) 8 months before presentation, with no prior evidence of *Streptomyces*.

The patient reported using an expired dialysate bag during his most recent exchange, performed independently in a temple setting. The dialysate had expired 5–7 months earlier and was stored at ambient temperature in a non–air-conditioned storeroom with intermittent sunlight exposure. Dialysate supplies were obtained through donation from other centers approximately 6 months before; several cartons were damaged on arrival, and stock organization lacked first-in, first-out rotation. Remaining supplies were inspected 2 weeks after hospital admission.

Dialysis exchanges were performed in a multipurpose room that also served as a sleeping area, with dialysis materials placed on the floor and no dedicated preparation space or hand hygiene facilities.

On presentation, the patient was afebrile and hemodynamically stable, with mild diffuse abdominal tenderness and a clean catheter exit site. PDE showed leukocytosis of 1709 cells/mm^3^ (73% neutrophils). Empiric intraperitoneal cefazolin (1 gm/d) and ceftazidime (1 gm/d) were initiated, later converted to intravenous administration due to ultrafiltration failure. By Day 3, the PDE cell count decreased to 543 cells/mm^3^. Gram stain demonstrated Gram-positive filamentous bacilli. Acid-fast and modified acid-fast stains were negative, and cultures remained negative at 48 hours. MALDI-TOF mass spectrometry misidentified the isolate as *Corynebacterium ureicelerivorans*.

**Figure 1:**
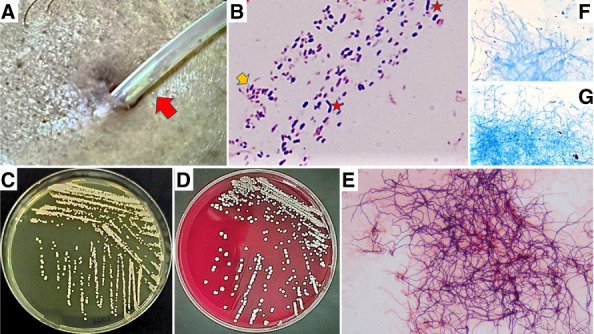
**Microbiologic and visual findings in a case of *Streptomyces olivaceus* peritonitis in a PD patient.** (A) External view of the PD catheter still *in situ*, showing dark cream-colored intraluminal colonization near the exit site (red arrow), suggestive of biofilm or filamentous bacterial growth. (B) Gram stain of intraluminal catheter material demonstrating filamentous Gram-positive bacilli arranged in tangled clusters (yellow arrow), with red stars indicating elongated, irregularly shaped bacillary forms, morphologically consistent with actinomycetes. (C) Tryptic soy agar and (D) blood agar demonstrating dry, wrinkled, chalky colonies of the isolate after 72 hours of aerobic incubation at 35°C; no hemolysis observed. (E) Gram stain from cultured colonies confirming abundant filamentous Gram-positive structures. (F) AFB stain and (G) modified AFB stain from cultured colonies, both negative, effectively excluding *Nocardia* species. AFB, acid-fast bacilli; PD, peritoneal dialysis.

Because of persistent symptoms, the PD catheter was removed on Day 8. Intraluminal inspection showed dark granular debris (Figure 1A). Gram stain of catheter material showed filamentous Gram-positive bacilli arranged in tangled clusters (Figure 1B). After 72 hours of aerobic incubation at 35°C, dry, wrinkled, chalky colonies with pale yellow pigmentation and no hemolysis grew on tryptic soy agar and blood agar (Figure 1, C and D). Gram stain from cultured colonies confirmed filamentous Gram-positive structures (Figure 1E), while acid-fast and modified acid-fast stains were negative (Figure 1, F and G), excluding *Nocardia*. Findings were consistent with *Streptomyces* species.^3,10^

On Day 34, 16S rRNA gene sequencing identified the organism as *S*. *olivaceus* with 100% identity and full query coverage over 686 base pairs (GenBank accession number OM131747.1).

The patient was treated with intravenous amoxicillin-clavulanate (1.2 gm every 8 hours) and trimethoprim-sulfamethoxazole (TMP-SMX; 10 mg/kg per day trimethoprim component, divided every 12 hours and adjusted for kidney failure). TMP-SMX was discontinued on Day 29 due to pancytopenia and replaced with oral amoxicillin-clavulanate (625 mg twice daily) and clindamycin (300 mg three times daily). Clinical symptoms improved steadily. On Day 63, paracentesis revealed exudative ascites with 317 cells/mm^3^ (93% mononuclear cells), with negative cultures and PCR assays. Antibiotics were discontinued on Day 96 after an 8-week course. The patient remained clinically well without recurrence as of February 6, 2026, and continued maintenance hemodialysis.

Postrecovery inspection of expired dialysate revealed visible defects, including debris between bag layers and particulate contamination. Cultures yielded multiple organisms, including *Pseudomonas oryzihabitans*, *Cellulomonas*, *Microbacterium*, *Penicillium*, and *Aspergillus*. Nonexpired dialysate from the same batch was unavailable for comparison.

## Discussion

This case highlights both a rare pathogen and an underrecognized risk factor in PD care: the use of expired dialysate. Although *Streptomyces* species are often considered contaminants, the filamentous Gram-positive organism identified in this patient was confirmed *via* 16S rRNA sequencing with 100% identity—establishing its clinical relevance. Diagnostic challenges were evident, as the organism was not detected by routine culture and was misidentified by MALDI-TOF, reflecting known limitations related to incomplete spectral libraries, underrepresentation of environmental organisms, and variability in protein expression.^3,11^ The 2022 International Society for Peritoneal Dialysis Guideline recommends molecular diagnostics in persistent culture-negative peritonitis, particularly in cases unresponsive to standard antimicrobial therapy.^12^

MALDI-TOF misidentification—often as *Corynebacterium* or other actinomycetes—has been reported, particularly when standard databases lack adequate representation of environmental organisms.^11,13^ Factors, such as colony age, culture conditions, and polymicrobial background, may further compromise accuracy, likely contributing to the initial misidentification in this case.^14^

Treatment of *Streptomyces* infections remains undefined, although susceptibility data support the use of aminoglycosides, carbapenems, and linezolid.^3^ Our patient responded favorably to amoxicillin-clavulanate and TMP-SMX (subsequently replaced with clindamycin due to pancytopenia), combined with prompt catheter removal and extended therapy—consistent with International Society for Peritoneal Dialysis principles for refractory or atypical infections.^12^

Crucially, this case highlights system-level vulnerabilities in PD programs, particularly in decentralized or home-based settings. The expired dialysate was used during an unsupervised exchange in a nonclinical environment. Although *Streptomyces* was not isolated from remaining bags, inspection revealed visible deterioration and polymicrobial contamination, including *P*. *oryzihabitans*, *Cellulomonas*, *Microbacterium*, *Penicillium*, and *Aspergillus*. These findings support that expired dialysate—especially when stored outside validated conditions—can serve as a conduit for environmental exposure, even without overt packaging defects.

Supporting this, the Check List to Improve Patient Self-care and Product Defect Report in Continuous Ambulatory Peritoneal Dialysis study in Thailand implemented a structured checklist enabling patients to inspect PD supplies.^15^ Patients identified defects such as staining, leaks, and unclear fluid in 0.2% of nearly 4000 bags, demonstrating the feasibility of patient-led surveillance. Although not yet universally adopted, national guidelines in Thailand already encourage such practices given their association with peritonitis risk.

From a public health perspective, this case underscores the need for strengthened supply chain oversight, prompt removal of expired stock, and patient education on product integrity. Regulatory guidance advises against the use of expired or compromised dialysate,^6^ yet real-world constraints often undermine these safeguards.

In conclusion, *S*. *olivaceus* peritonitis is a rare but clinically significant cause of PD-associated infection. This case highlights the limitations of conventional diagnostics, the value of molecular identification, and the risks associated with expired dialysate in home-based PD. Strengthening supply chain and patient-level safety practices will be essential as PD expands globally.

## Data Availability

Original data generated for the study will be made available upon reasonable request to the corresponding author. Data Type: Health Care Data; Image Data. Reason for Restricted Access: The data that support the findings of this study are not publicly available due to the privacy of research participants but are available from the corresponding author upon request.

## Declarative Statements

This study complies with the Declaration of Helsinki.
